# Integrating conductive electrodes into hydrogel-based microfluidic chips for real-time monitoring of cell response

**DOI:** 10.3389/fbioe.2024.1421592

**Published:** 2024-08-27

**Authors:** Ayda Pourmostafa, Anant Bhusal, Niranjan Haridas Menon, Zhenglong Li, Sagnik Basuray, Amir K. Miri

**Affiliations:** ^1^ Department of Biomedical Engineering, Newark College of Engineering, New Jersey Institute of Technology, Newark, NJ, United States; ^2^ Department of Mechanical Engineering, Rowan University, Glassboro, NJ, United States; ^3^ Department of Chemical Engineering, Newark College of Engineering, New Jersey Institute of Technology, Glassboro, Newark, NJ, United States

**Keywords:** conductive ink, bioprinting, microfluidic chip, real-time screening, spheroids

## Abstract

The conventional real-time screening in organs-on-chips is limited to optical tracking of pre-tagged cells and biological agents. This work introduces an efficient biofabrication protocol to integrate tunable hydrogel electrodes into 3D bioprinted-on-chips. We established our method of fabricating cell-laden hydrogel-based microfluidic chips through digital light processing-based 3D bioprinting. Our conductive ink includes poly-(3,4-ethylene-dioxythiophene)-polystyrene sulfonate (PEDOT: PSS) microparticles doped in polyethylene glycol diacrylate (PEGDA). We optimized the manufacturing process of PEDOT: PSS microparticles characterized our conductive ink for different 3D bioprinting parameters, geometries, and materials conditions. While the literature is limited to 0.5% w/v for PEDOT: PSS microparticle concentration, we increased their concentration to 5% w/v with superior biological responses. We measured the conductivity in the 3–15 m/m for a range of 0.5%–5% w/v microparticles, and we showed the effectiveness of 3D-printed electrodes for predicting cell responses when encapsulated in gelatin-methacryloyl (GelMA). Interestingly, a higher cellular activity was observed in the case of 5% w/v microparticles compared to 0.5% w/v microparticles. Electrochemical impedance spectroscopy measurements indicated significant differences in cell densities and spheroid sizes embedded in GelMA microtissues.

## 1 Introduction

High-throughput screening platforms have been used for drug optimization, screening, and toxicology testing (Rothbauer et al., 2018; Bhusal et al., 2024). Conventional screening platforms allow the study of cell-cell and cell-drug interactions on a large scale; however, these platforms require expensive robotic tools for liquid handling and data analysis (Li et al., 2014). In addition, the conventional platforms do not allow the incorporation of three-dimensional (3D) microenvironment and physiological conditions (Griffith and Swartz, 2006; Li et al., 2014). Current advances in miniaturization have allowed the application of microfluidics to overcome limitations possessed by conventional platforms (Cavo et al., 2016).

3D structural features were introduced within microfluidic systems using extracellular matrix (ECM) scaffolds and cell organizations mimicking the native architecture and environment of human physiology (Yesil-Celiktas et al., 2018). Conventional or additive manufacturing methods, along with biomimetic biomaterials, have been employed to create microtissues and 3D architectures (Yesil-Celiktas et al., 2018; Dogan et al., 2022; Bhusal et al., 2024). Regulating such microtissues or organoids can be challenging in a microfluidic setting, which calls for label-free monitoring and real-time sensing of cells (Moysidou et al., 2021; Pitsalidis et al., 2021). Real-time tracking of biological activities and cellular interaction processes allows for regulating biochemical parameters to improve control over cell responses (Moysidou et al., 2021). The current fabrication approaches for microfluidic devices having integrated electrodes require a post-fabrication process, which is cost-ineffective, labor-intensive, and requires sequential integration (Wang et al., 2018; Miri et al., 2019; Shin et al., 2019).

Recent research shows the role of electrodes for deoxyribonucleic acid (DNA) analysis, cell handling, chemical analysis detection, processing, separation-based detection, and other applications (Erickson and Li, 2004; Hiramoto et al., 2019). For example, transducers packed within the electrodes in a microfluidic channel have been applied to detect cancer biomarkers rapidly (Cheng et al., 2021). A microfluidic platform was designed to manipulate and separate microparticles and live cells, such as red blood cells, from sickle cells and bacteria, which used particle size, shape, and elasticity (Kose et al., 2009). Conventional devices use polydimethylsiloxane (PDMS) or plastic molding for encasing both cell-laden parts and electrodes, possess limitations of weak functionality in mimicking physiological environments of target tissues (Shen et al., 2019), and require post-fabrication, making the process length complex and cost-inefficient (Yesil-Celiktas et al., 2018). Hallfors *et al.* fabricated novel liquid metal electrodes in a PDMS microfluidic device by injecting when PDMS and glass are covalently bounded for the neural simulation (Hallfors et al., 2013). The interdigitated gold electrodes were prepared by sputter coating, patterned by standard photolithography technique, and covered with PDMS channel to separate targeted cells using dielectrophoresis-based micro separators (Dalili et al., 2021).

Integrated electrodes can also be used for cell stimulation. In the case of cardiomyocytes, electrical stimulation the alignment, electrical coupling, and cell growth (Wang et al., 2020). We can provide cues that promote cardiomyocyte alignment by designing and fabricating substrates with specific surface features, such as microchannels or patterned structures (Gong et al., 2019). In-line electrical stimulation and recording help the maturation/differentiation of cells and monitor their responses.

The common conductive materials for engineered electrodes are metal- and carbon-based materials (Hamzah et al., 2018). Their applications are limited because of their biocompatibility or narrow electrochemical potential window, reducing the sensing scope for metal-based electrodes. Poly-(3,4-ethylene-dioxythiophene)-polystyrene sulfonate (PEDOT:PSS) has gained attention from researchers because of its thermal stability, oxidative stability, and electrochemical stability (Kara and Frey, 2014; Pitsalidis et al., 2022). As an electrochemical sensor, PEDOT:PSS has easily modified physicochemical properties and biocompatibility and allows easy surface modification and functionalization, retaining electrical conductivity (Benoudjit et al., 2018). The PEDOT:PSS mixed with polyethylene glycol diacrylate (PEGDA) was 3D printed using stereolithography to deliver electric stimulation to enhance neural differentiation (Heo et al., 2019).

**FIGURE 1:**
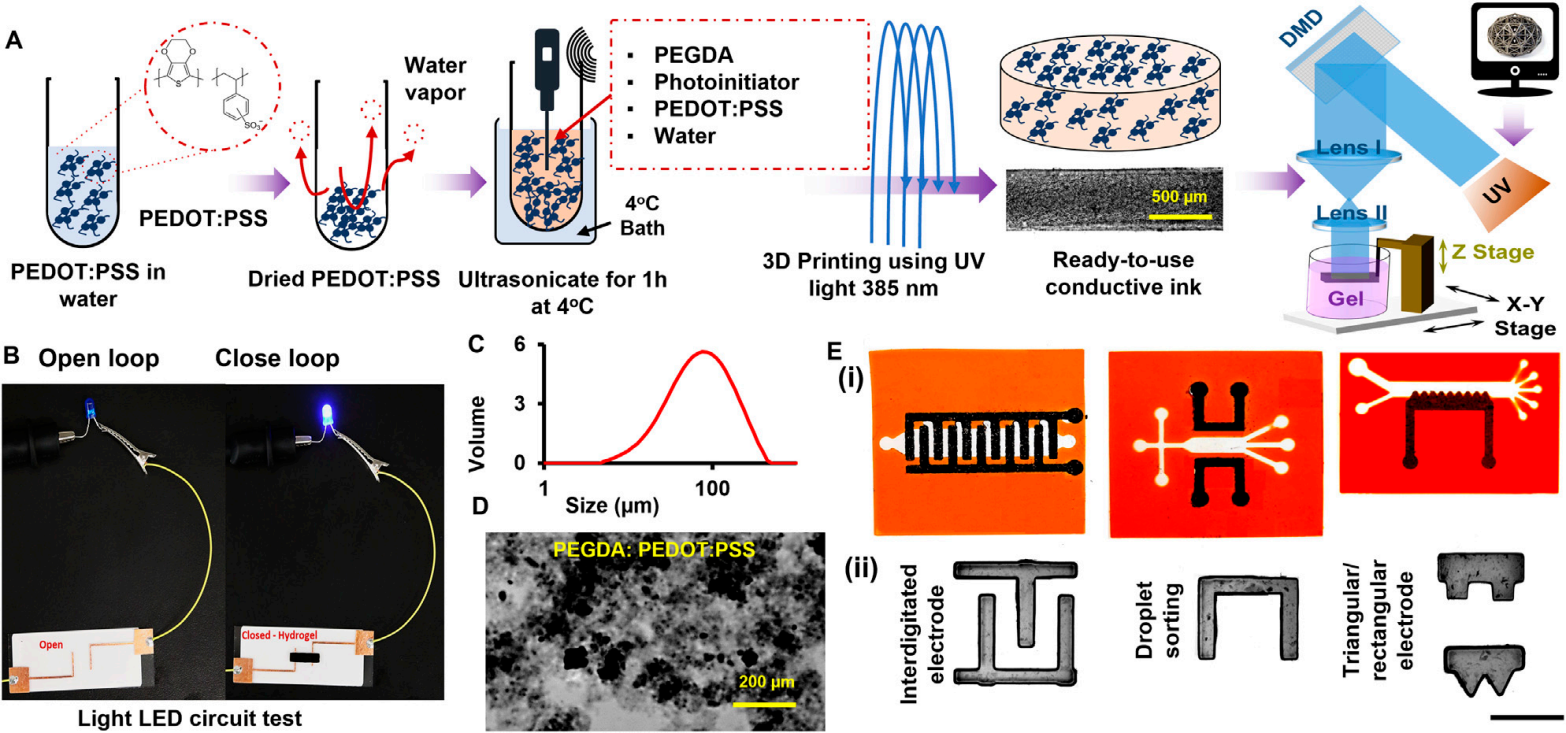
**(A)** Preparation process of conductive hydrogel doped with PEDOT:PSS—commercially available PEDOT:PSS is freeze-dried and mixed with PEGDA and photoinitiator at set concentrations and ultrasonicated for 1 hr at 4°C water bath which can be 3D printed to get conductive structure, **(B)** Image of lighting a LED light using closed loop (right) and open loop (left) with and without conductive hydrogel, respectively, **(C)** Particle size distribution, **(D)** Brightfield image of the produced particles before embedding into ink, and **(E)** Designed conductive microfluidic chip on the CAD file ii) Printed samples using conductive ink for different applications (Scale: 1 mm).

Combining 3D printing technology and conductive hydrogel-based ink enables the seamless integration of electrodes into a microfluidic platform, eliminating the need for post-processing steps in biochemical analysis. Digital Light Processing (DLP) forms structures layer by layer by solidifying photocurable materials with projected ultraviolet (UV) patterns, which is crucial for creating features in hydrogel electrodes (Han et al., 2024). The DLP method provides high-resolution capabilities that surpass conventional techniques such as inkjet and screen printing, which are limited by low resolution and complex procedures. DLP allows for the precise creation of intricate micro-scale structures necessary for practical electrode function. Unlike traditional methods, DLP can fabricate detailed, conductive hydrogel scaffolds efficiently. Wu et al. demonstrate that DLP printing with interfacial polymerization produces conductive GelMA-Pani hydrogels with improved electrical properties and biocompatibility, making it an ideal choice for advanced electrode fabrication (Yuk et al., 2020). With more control over the shape fidelity, we have more control over PEDOT:PSS microparticle sizes. We intended to apply our DLP printing platform to create hydrogel-based conductive electrodes for microfluidic devices, eliminating the need for post-processing for biochemical analysis (Bhusal et al., 2021; Bhusal et al., 2022). Our inks include gelatin methacryloyl (GelMA) and PEGDA. At the same time, PEDOT:PSS-doped PEGDA will be our conductive ink (see Figure 1). We accessed the printability of PEDOT:PSS-doped PEGDA at different mass concentrations by creating hydrogel electrodes. Physical properties, such as elastic modulus and swelling behavior, and electrical properties, such as conductivity and impedance, were evaluated using standard methods. We measured the electrochemical impedance spectroscopy (EIS) for different cell settings in the cell-laden part of the microfluidic model.

## 2 Experimental section

### 2.1 Bioprinting process

Using a custom-built multi-material DLP system established in our lab (Bhusal et al., 2021), printing parameters for a 100 µm layer were used to print at different PEDOT:PSS concentrations (0, 0.5, 1, 1.5, 3, and 5%), while UV exposure time was optimized based on a UV light system (Visitech; Wetzlar, Germany). The wavelength of the UV light system is ∼380 nm and uses the light intensity of ∼0.7 W cm^−2^ at the focal plane. A predefined computer-aided design (CAD) model of the target structure was prepared and sliced layer-by-layer. For the single material, the build platform was lowered using a linear *z*-axis platform, and layer-by-layer crosslinking was performed by sequentially raising the platform. The program selected the bioink that covers a smaller space or dispersed pattern for interposed multi-material. After the washing step, it moved to another area, and this process continued. The bioprinting process, UV exposure time, and the model variations were explained previously (Bhusal et al., 2021).

### 2.2 Conductive ink and bioink preparation

GelMA was synthesized and prepared to make cell-laden bioink. PEGDA (Mn = 700, Sigma-Aldrich, MO) was used as the ink for the support structure. PEDOT:PSS (0%–5.0% w/v) doped in PEGDA for conductive hydrogel ink. The GelMA was synthesized following an established protocol (Miri et al., 2018) based on porcine skin gelatin (Sigma-Aldrich, St. Louis, MO) and methacrylic anhydride (MA, Sigma Aldrich). The gelatin solution (10% w/v) was prepared in Dulbecco’s phosphate-buffered saline (DPBS), and methacryloyl was induced by adding MA at 5% v/v. The solution was dialyzed for 1 week and lyophilized to obtain the GelMA. The bioreactor material is prepared by mixing GelMA (5% w/v) in DPBS and 0.2% w/v lithium phenyl-2,4,6-trimethyl-benzoyl-phosphinate (LAP, Sigma-Aldrich) and stirred at 40°C until homogenous a transparent mixture is achieved. The maximum light absorbance occurs around 375 nm for the LAP (Fairbanks et al., 2009), and the custom-built DLP printer operates at 380 nm, ensuring an optimal photocuring process. The second ink was prepared by mixing PEGDA 30% v/v in DPBS with 0.1% LAP and ∼1% w/v gel-based commercial orange food dye (AmeriColor, Placentia, CA). We used commercially available ingredients for PEDOT:PSS. It was initially lyophilized at − 50°C for 48 h to obtain the PEDOT:PSS microparticles. They were mixed with DPBS and 0.1% PI before being added to the PEGDA precursor to make a 30% v/v solution. The obtained mixture was sonicated for ∼1 h in an ice bath to get a homogenous mixture of microparticles and gel precursors.

### 2.3 Structural characterization

The swollen hydrogels were frozen and lyophilized for scanning electron microscopy (SEM). The lyophilized samples were cut, and their cross-sections were coated with platinum using a turbo sputter coater (EMITECH, K575X) and before SEM imaging (JSM-7900F Schottky Field Emission Scanning Electron Microscope). We used a rotating angle dynamic light scattering (DLS) machine (Malvern Mastersizer 3000) for the measurements. The data are shown in Figure 1C.

### 2.4 Physical characterization

The mechanical stiffness of samples was measured using a compression test to determine the structural stability. Cylindrical samples were prepared (diameter 4 mm, 3 mm height, out of 100 µm layers; exposure time of 0.6 s) and placed in a mechanical tester (Shimadzu EZ-SX, Columbia, MD). The displacement-controlled test was performed at a 1 mm/min strain rate. In addition, the swelling ratios of samples were measured to determine the structural fidelity of the samples. The samples were submerged in phosphate-buffered saline (PBS) at 37°C for 24 h, and swelling weight was measured. The PEDOT:PSS-doped PEGDA samples were lyophilized before measuring the dry weight. The swelling ratio was evaluated as (swelling weight - dry weight)/dry weight. Furthermore, rheology measurements were made using a Waters HR10 Rheometer (TA Instruments, Delaware) in a parallel-plate mode and two modes: a frequency sweep (0.01–100 Hz) at 150 μm separation and viscosity-shear rates. The data were reported in two formats: elastic shear modulus G′ and viscous shear modulus G″ vs. oscillation frequency, and dynamic viscosity vs. shear rate (1/s), as summarized in Supplementary Figure S2.

### 2.5 Conductivity measurement

The conductivity of our conductive ink was measured using the four-probe method. The current passed through the outer probe causes a reduction in the potential between the two inner voltage sensing probes that can be applied to measure the resistance of the sample [Resistance (*R*) = Voltage (*V*)/Current (*A*)]. The value of resistance is the resistivity of the material times cross-section area (*A*) divided by the length of the 3D printed material (*L*) [Resistivity (*ρ*) = Resistance (*R*) x Area (*A*)/Length (*L*). If the resistivity of the material is known, then the conductivity of the material may be calculated as the reciprocal of the resistivity [Conductivity (*σ*) = 1/Resistivity (*ρ*)].

### 2.6 Electrochemistry characterizations

The EIS measurements were performed using the Agilent 4294A Impedance Analyzer from Keysight Technologies. The cyclic voltammetry (CV) signals were obtained using a Gamry (Reference 600+) potentiostat. The conductive and non-conductive hydrogels were fixed to a gold microelectrode surface, acting as the conducting interface between the microelectrodes and the GelMA ink. The EIS measurements were performed in the frequency range from 1 kHz to 1 MHz. The applied voltage is 100 mV. The EIS signals were recorded once the response from the samples was equilibrated. All measurements were performed at room temperature.

### 2.7 Biocompatibility

A semi-circular geometry of the conductive material was crosslinked in 24 well-plates, and human-derived mesenchymal stem cells (ATCC) were seeded in the well with 50,000 cells/mL. In each well, 1 mL of additional media was added and cultured in an incubator for 24 h at 37°C. After 24 h, the samples were collected, and a quantitative cell assay was performed using Cell Proliferation Kit II (XTT, Cell Viability Kit, Cell Signaling Technologies) and lactate dehydrogenase (LDH, Cytotoxicity Detection Kit, Roche) measured by absorbance of light at 450 nm and 490 nm respectively. Additionally, the samples on the well-plate were further subjected to live/dead testing using a standard live/dead staining kit (PromoKine Live/Dead staining kit II, Heidelberg, Germany). They were imaged using a Nikon fluorescent microscope (Nikon, Melville, NY).

### 2.8 Tumor spheroid assay

Monoculture breast cancer (MDA-MB-231, ATCC) spheroids were prepared with an initial seeding density of 1×10^4^ cells/well in 200 cell suspension seeded to a non-adherent round-bottom 96-well-plate (Corning, NY, United States). The ultra-low attachment (ULA) plates were centrifuged at 2,500 rpm/850 xg for 5 min in a plate centrifuge device (VWR, Radnor, PA, United States) to form spheroids through centrifugal force. At Day 3, we started quantifying spheroids’ diameters and roundness using ImageJ (NIH). After assessment, cellular micro-complexes were encapsulated into different GelMA solutions and DLP-printed for the EIS measurement.

### 2.9 Statistical analysis

Results were expressed as mean ± standard deviation. Each experiment was repeated at least twice to confirm reproducibility. Statistical analysis was performed using a one-way ANOVA with a Tukey *post hoc* adjustment for pairwise comparisons. Statistical significance was set at *p* < 0.05. Statistical analyses were conducted using R within the RStudio integrated development environment.

## 3 Results and discussions

### 3.1 Physical characterization of conductive ink

The fabrication of PEDOT:PSS microparticle-doped PEGDA was optimized by varying selected ink parameters, such as mass concentrations and dope ratio (Figure 1A), while showing some examples in Figure 1E, including interdigitated electrodes, droplet sorting, and triangular/rectangular electrodes. We found the optimum LAP concentration to be around ∼1% w/v, proper PEGDA concentration around 30% v/v for high microparticle concentration, practical printing layer thickness around 100 μm, and light exposure time (<0.6 s per layer) for the desired pattern fidelity and structural stability with our DLP bioprinter (Bhusal et al., 2021). Our preliminary data showed that higher PEGDAm concentrations (i.e., 40%–80% v/v) could lead to a lower threshold for microparticle-to-PEGDA ratio, while lower backbone concentrations (i.e., 10%–25% v/v) yield unstable electrodes, making them unsuitable for our application (data not shown). Although the mass concentration of the selected conductive polymer has been limited to 1% w/v for different polymers, including PEGDA (Testore et al., 2022), our protocol is able to make microparticle concentration at 5% w/v with suitable physical quality, including elasticity and toughness, and potential applications to complex geometries, including sharp edges and curved channels.

**FIGURE 2:**
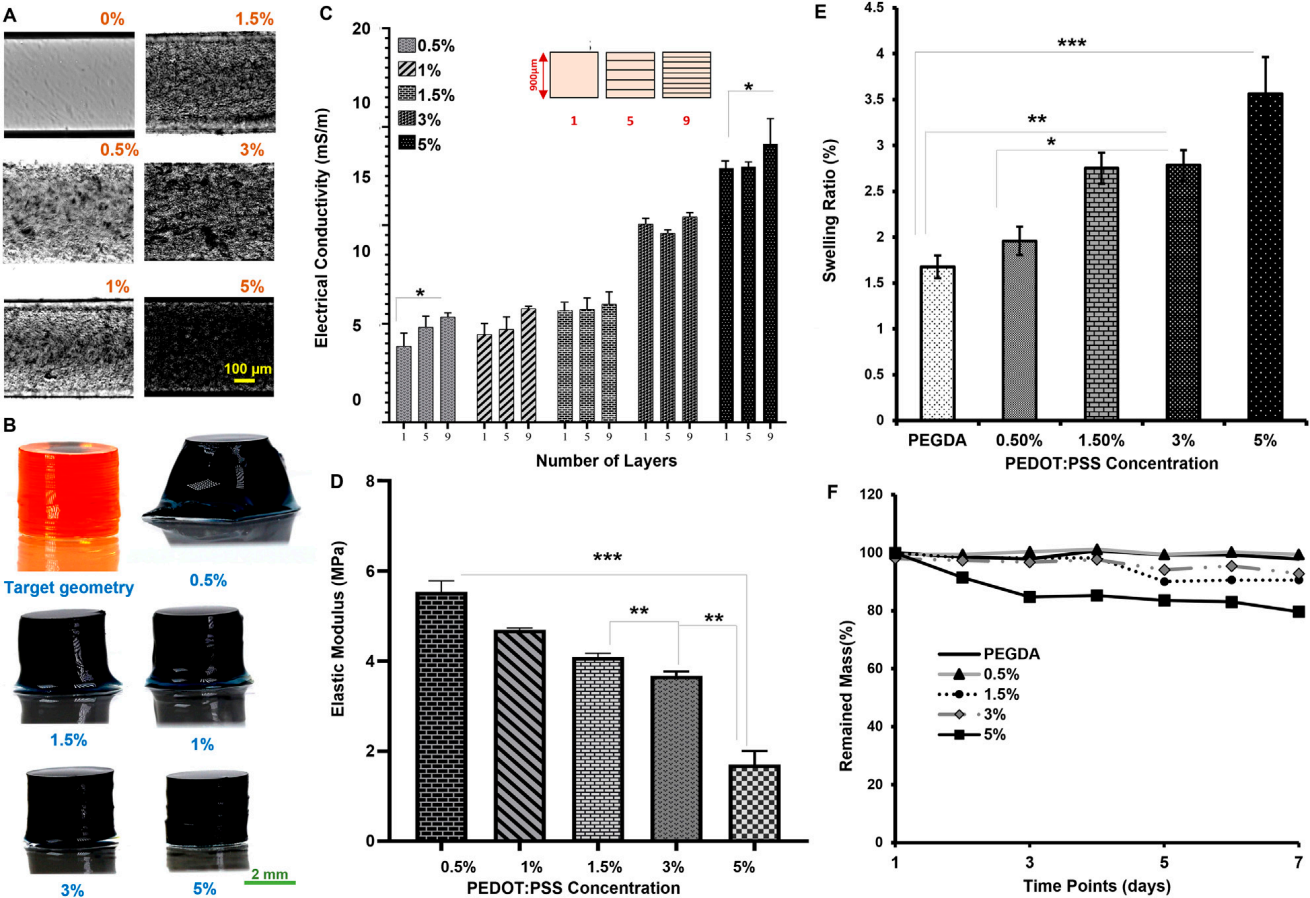
Optical characterization: **(A)** Gray scale imaging of different concentration of PEDOT:PSS (0, 0.5, 1, 1.5, 3, and 5%) in 30% PEGDA printed one-layer (Scale:100 µm); **(B)** (perspective view) 3D optical imaging of different concentration of PEDOT:PSS at 0.6 s UV exposure each layer [Scale: 2 mm]; **(C)** Conductivity based on the number of layers in the process; **(D)** Mechanical characterization. Stiffness at different PEDOT:PSS concentration; **(E)** Swelling ratio of PEGDA doped with PEDOT:PSS particles at 24 h; **(F)** Degradation of samples over time. (*p* > 0.05, *p ≤ 0.05, **p ≤ 0.01, ***p ≤ 0.001), *p* < 0.05 represent significative differences for all other comparisons. All data are presented as the mean ± SD (n = 4).

The PEDOT:PSS microparticles were observed to be between 50 μm and 120 µm (Figure 1C), more than the typical pore size of undoped PEGDA, considerably less than 50 µm (Bhusal et al., 2022). This size difference ensured the biophysical stability and entrapment of the microparticles within the hydrogel network (Lim et al., 2021). The homogeneity of microparticle distribution was also assessed by grayscale imaging for single layers in Figure 2A and color imaging for 3D samples in Figure 2B. The microparticles in the hydrogel block the UV light, resulting in higher UV penetrations, which can be the main limitation for choosing the concentration of our conductive co-polymer in the literature (Testore et al., 2022).

The ink opacity means a lower optical refractive index, which indicates the light absorption by the ink. The impact of this opacity was assessed by measuring conductivity for layer number from 1 (900 µm-) layer to 9 (100 µm-) layers towards a consistent shape (Figure 2C, statistically significant). The lowest conductivity (∼4 m/m) was observed in the case of 0.5% w/v. In contrast, the highest conductivity (∼14 m/m) was recorded at 5% w/v. There are no significant changes in the values for selected layer numbers. The UV curing effect is highlighted at lower microparticle concentrations by reducing conductivity for a thick layer. Above 1.5% w/v, the effects are minimal, and the layer-by-layer printing would be less affected. The impact of microparticles on the ink was further verified via bulk elastic modulus in Figure 2D and swelling ratio (i.e., the ability of the backbone to absorb water) in Figures 2E, F.

The elastic modulus indicates that the stiffness is reduced by more than 50% when increasing the microparticle concentration from 0.5% to 5% w/v, in which the minimum modulus recorded is 1.7 ± 0.30 MPa for 5% w/v. The elastic modulus is 5.5 ± 0.25 MPa for 0.5% w/v, as shown in Figure 2D. The decreased stiffness (see also shear moduli in Supplementary Figure S2) impacts the ability of hydrogels to expand in volume, which can be quantified through the swelling ratio. The swelling ratio at 24 h post-fabrication, in Figure 2E, was found to be 1.69 ± 0.16 for the case of 0.5% w/v and 3.56 ± 0.42 for the case of 5% w/v. A higher crosslinking density leads to a lower swelling ratio. This observation may suggest using microparticle concentration lower than 3% w/v for electrical electrodes. The light energy absorption by the microparticles limits the formation of stronger bonds within PEGDA (Supplementary Figure S3). The mass degradation testing confirmed the stability of our conductive inks for up to 1 week above 90%, except in the case of 5% w/v, as shown in Figure 2F.

### 3.2 Ink conductivity

**FIGURE 3:**
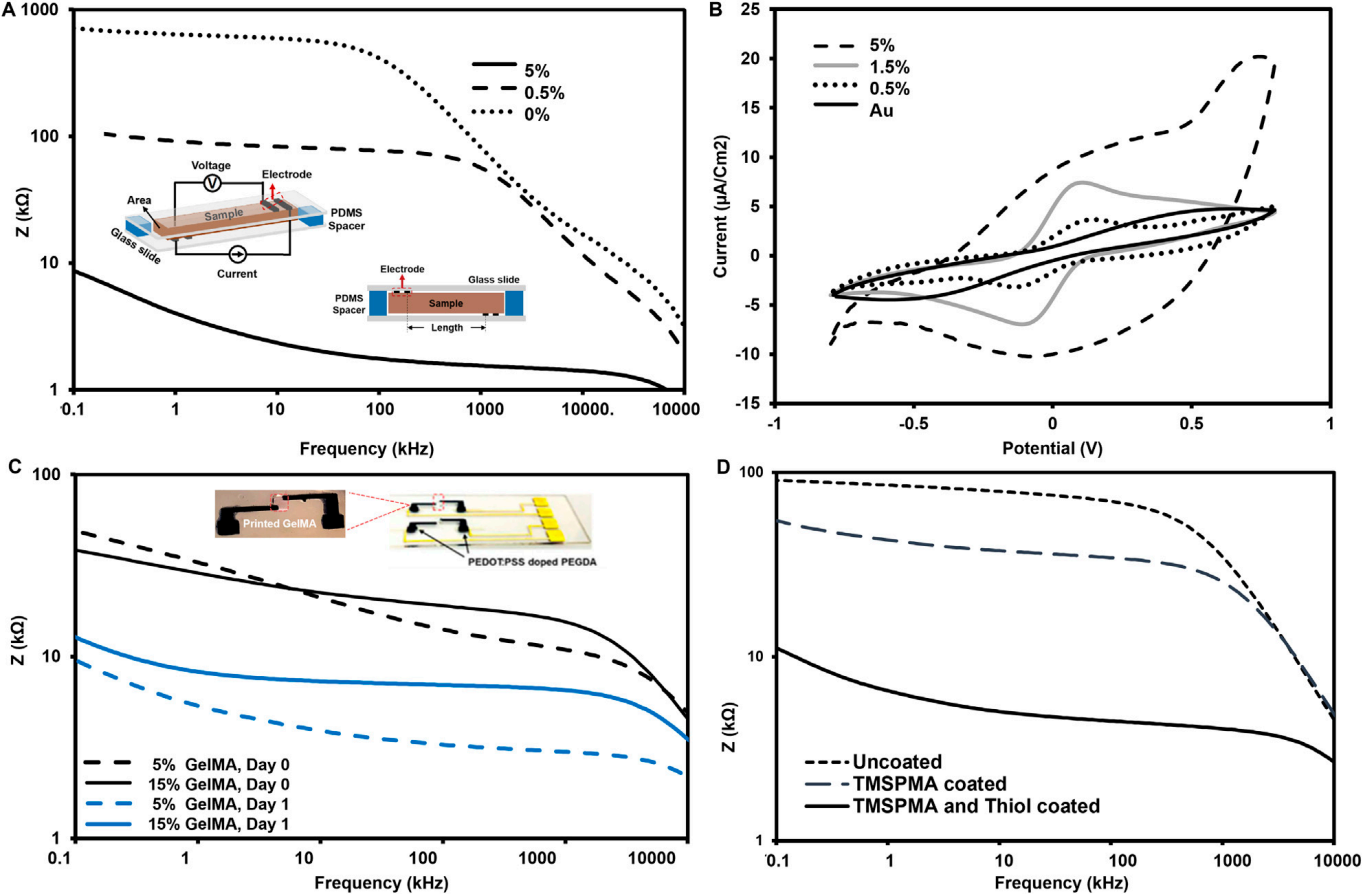
**(A)** Electrochemical impedance spectroscopy (EIS) curves of the selected concentrations for PEDOT:PSS particles in the hydrogel electrode covered Au electrodes, increase in signal-to-noise ratio. **(B)** CVs for Au electrode and PEDOT:PSS coated Au at 50 mV/s scan rate, in equimolar 5 mM K3 [Fe(CN)6]/K4 [Fe(CN)6] in 0.1 M KCl (the dots: the experimental data. **(C)** Bode plot showing impedance curves for measured the GelMA (5, 10, 15% wt) before and after swelling using 5% PEDOT:PSS hydrogel measurement electrodes. **(D)** Plot spectra of hydrogel-based electrodes of to compare coating effect on Au electrode slide.

The electrical conductivity of 3D printed microparticle-PEGDA samples is presented in Figure 3A, in which the insets show the set-up. Two extreme concentrations in the range of PEDOT:PSS microparticles 0.5%–5% w/v were used to show the variation of impedance as a function of frequency in the EIS measurements. Lower *Z* values indicate higher conductivities, while PEGDA (no microparticle) should be a low-conductivity case. A sharp drop in *Z* values after 100 kHz suggests that the measurement for frequencies higher than 100 kHz is dominated by the buffer (not the polymeric backbone). The doped PEGDA changed from 0.5% to 5% w/v improved conductivity at several orders of magnitude. The low- and high-frequency values are within one order of magnitude. The conductive ink with low impedance enhances the signal-to-noise ratio of impedance sensing and charge injection efficiency during EIS recording (Kim et al., 2022).

We used CV measurements to compare the redox efficiency of our electrodes vs. conventional interdigitated Au (see Figure 3B). Two broad peaks were observed for Au, including an anodic peak potential of around +0.5 V and a cathodic peak potential of around – 0.5 V. These peaks indicate a redox reaction. In the case of microparticle-doped PEGDA, the anodic peak is located at around +0.1 V, and the cathodic peak is located at around – 0.1 V. Regarding the considerable decrease in the peak-to-peak separation (∆E), the presence of microparticles at 0.5% and 1.5% w/v reduces the barrier for electron transfer, resulting in narrow ∆E and high peak currents. The further increase in the microparticle content to 5% w/v led to a different response, indicating a disrupted ion transfer during redox. This may further suggest that microparticle concentrations above 1.5% w/v can interrupt the microstructure of the PEG hydrogels, as noted in the literature (Lim et al., 2021).

The next phase was to measure the EIS for GelMA ink connected to 5%-microparticle-PEGDA electrodes. The EIS data for the GelMA between the two electrodes has been analyzed in Figure 3C. The presence of the middle part increased the *Z* values in Figure 3A compared to those in Figure 3C. The swelling ratio of the gelatin scaffold increased the buffer volume, thus reducing the *Z* values to numbers much closer to the *Z* values of pure electrodes. The last question on how the adhesion of the gel electrode can impact the *Z* values in Figure 3D indicates how the surface modification can ensure the consistency of the measurement by ensuring a seamless connection between sample and electrodes. This is important as the gel tends to swell post-fabrication, and this swelling (also seen in Supplementary Figure S4) can lead to separation from the gold electrodes.

### 3.3 EIS monitoring of cell-laden models

EIS sensing techniques (e.g., ECIS) are well established, and they rely on works in 2D cultures because cells attach to the electrodes. 3D cultures require the presence of scaffolds or suspended cell clusters (Voiculescu et al., 2020). Integrating conductive ink to print electrodes in contact with the cells in GelMA enabled us to record cell density in 3D. In our first experiment, the cell-laden GelMA samples are at different cell densities, from 0, 0.5, 1, 2, and 3 × 10^6^ cells/mL (Supplementary Figure S5). The Nyquist curves are shown in Supplementary Figure S5B, and the proposed equivalent circuit in Supplementary Figure S5A shows **R1** as the resistance from the device and external circuit. **R**_**ct**_ is the charge transfer resistance associated with the electrons’ transfer between the two microelectrodes. Instead of an ideal double-layer capacitance, the constant phase element **CPE1** is employed due to the inhomogeneity of the interface between the conductive gel and the microelectrodes. **CPE2** represents the impedance to the diffusion of electrons. **R**_**ct**_ and **CPE2** parallel **CPE1** as co-occurring phenomena (Shoar Abouzari et al., 2009; Ding et al., 2017). **R**_**ct**_ varies linearly with and increases cell density in the GelMA. This attributes the increase in the resistance to the flow of electrons through the cell-laden GelMA to the transepithelial electrical resistance of the cells (De León et al., 2020). The exponent value of **CPE1** is near one, indicating that the interfaces behave almost like ideal capacitances. The exponent values of the **CPE2** are close to 0.5, reflecting the porous nature of the conducting polymer matrix onto which the charges flow from the electrode.

**FIGURE 4:**
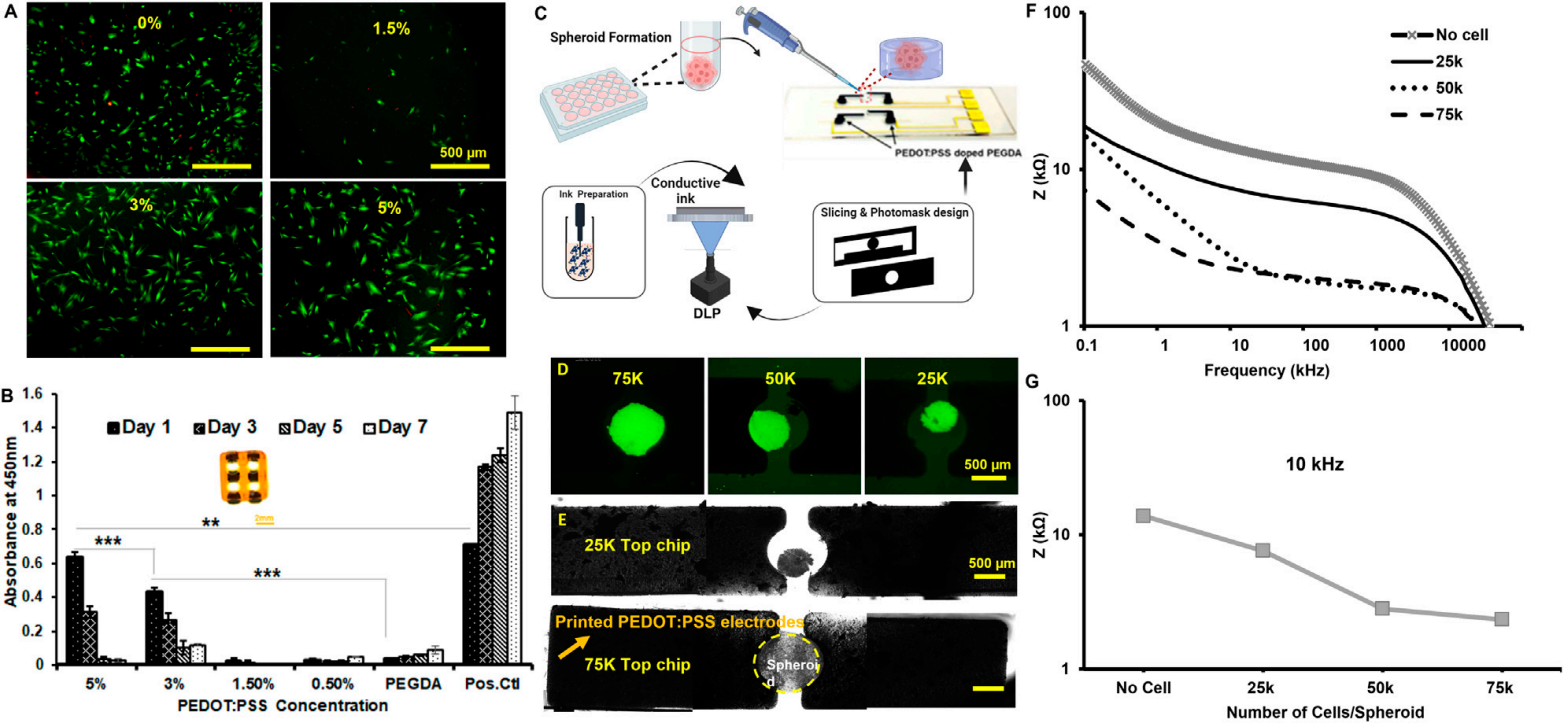
**(A)** Fluorescence image of live-dead staining of C2C12 cells on Day 1. **(B)** Comparison of cell viability by CCK8 assay for different concentrations. **(C)** Schematic of design of a hydrogel-based 3D printed electrode microfluidic screening of the spheroid size. **(D)** Fluorescent images of GFP-tagged MDA-MB-231 spheroids of different sizes (Scale: 500 µm). **(E)** Brightfield images of breast cancer (MDA-MB-231) spheroids of different sizes positioned in between the hydrogel electrodes in 2x objective. The scale bar is 500 µm. **(F)** The impedance magnitude for different spheroids of different sizes encapsulated in 5% GelMA. **(G)** Impedance comparison for different spheroid sizes at 10 kHz. comparison for different spheroid sizes at 10 kHz. Note that EIS measurements were carried out in the absence of a cell culture medium.

The live/dead imaging of cell seeding onto samples showed the biological response, and the quantitative metabolic activity of the conductive ink revealed the behavior for a network-like construct (see the inset image in Figures 4A, B). The kit showed increased cellular metabolic activity in higher concentrations (e.g., 3%–5% w/v), making our protocol appealing for the application of 3D biosensing in microfluidics (we observed a significant seeding capacity at 3%–5% w/v). The cellular activity was reduced over 1 week for all conductive ink groups, compared to the 2D culture; however, the metabolic activity of 3%–5% w/v up to Day 3 makes them suitable for 3-day applications, such as the example in Figures 4C, D. The electrode made by 5% w/v of microparticles showed how the cell spheroids size by varying cell density, 25K–75K per spheroid, can be monitored by EIS measurements. The Nyquist curves distinguish the difference between the control and the selected cell spheroids in Figure 4E. The Bode plot in Figure 4F further reveals the EIS variations based on cell density, and the selected frequency shows the drop in the impedance. For the EIS measurements, we used similar-sized electrodes to monitor tumor cell spheroids. The size of the spheroid increases with a rise in the cell number, as shown in Supplementary Figure S7. The rise in the spheroid size increases the contact area between the spheroid and the electrode, causing a decrease in the impedance (Figure 4F). It should also be noted that as the cell number increases, the amount of 3D-printed GelMA hydrogels in the spheroid is the same volume. Figure 4F shows the highest impedance in the case of acellular samples, as the amount of GelMA in the spheroid decreases and the contact area increases. Thus, the cell membranes may offer a low-resistance pathway through the contact area between the cells and electrodes (Schmid et al., 2016). We also compared the case in Figures 4C, D with a case of 2D (no gel electrode), and the results are summarized in Supplementary Figure S7.

## 4 Concluding remarks

Incorporating conductive hydrogels opens a broad spectrum of tools for real-time analysis of biological agents and their responses within 3D environments. Our previous work established that we may produce multiple bioreactors or cell-laden parts in a single microfluidic chip [22], which can be further enabled by incorporating hydrogel-based electrodes into such chips. The approach allows the rapid integration of conductive electrodes in microfluidic devices, eliminating the need for post-processing. This work establishes the groundwork for creating 3D-printed hydrogel-based electrodes in microfluidic devices in a seamless fabrication process. The cell-laden part models disease, organ, or tissue that may allow biochemical analysis throughout EIS or any similar mechanisms (Magar et al., 2021). Future work can target the classification of EIS data for specific scenarios.

The electrodes can induce electrical stimulation to control cell orientations, morphology, and gene expressions. Electrical stimulation and recording can improve the contraction functionality of myoblasts or cardiomyocytes (Kitsara et al., 2019). The stimulation can modulate their gene expressions, metabolisms, and calcium handling, affecting their contractile properties. Integrating electrical stimulation and impedance spectroscopy presents a powerful approach for investigating cardiac cell contraction. By monitoring impedance changes during stimulation, valuable insights can be gained into the mechanical properties and contractile behavior of cardiac cells or tissue. Impedance spectroscopy facilitates the detection of impedance magnitude and phase alterations, providing valuable information about changes in cell morphology, cell-cell coupling, and ECM stiffness. Classifying the meaningful interpretation of impedance changes concerning cardiac contractions can be a potential future direction.

## Data Availability

The original contributions presented in the study are included in the article/Supplementary Material, further inquiries can be directed to the corresponding author.
