# Supplementary material for: Integrating conductive electrodes into hydrogel-based microfluidic chips for real-time monitoring of cell response
Source: Front Bioeng Biotechnol. 2024 Aug 27;12:1421592. doi: 10.3389/fbioe.2024.1421592 (PMC11384590; doi:10.3389/fbioe.2024.1421592)
Supplement: Supplementary file 1 [file DataSheet1.PDF]

Supplementary Material

Integrating conductive electrodes into hydrogel-based microfluidic chips for real-time monitoring of cell response

Authors: **Ayda Pourmostafa[1†], Anant Bhusal[2†], Niranjan Haridas Menon[3], Zhenglong Li[3], Sagnik Basuray[3], Amir K. Miri[*1]**

Affiliations:

[1]Department of Biomedical Engineering, Newark College of Engineering, New Jersey Institute of Technology, 323 Dr Martin Luther King Jr Blvd, Newark, NJ 07102, USA;

[2]Department of Mechanical Engineering, Rowan University, 201 Mullica Hill Rd, Glassboro, NJ 08028, USA;

[3]Department of Chemical Engineering, Newark College of Engineering, New Jersey Institute of Technology, 323 Dr Martin Luther King Jr Blvd, Newark, NJ 07102, USA.

[†] *Equal Contributions*

[*]*Corresponding author:*

Dr. Amir K. Miri, *PhD*

e-mail: am3296@njit.edu; Phone: 973-596-6366

Postal Address: 323 Dr Martin Luther King Jr Blvd,

Fenster Hall 624 (BME), Newark, NJ 07102-1982, USA

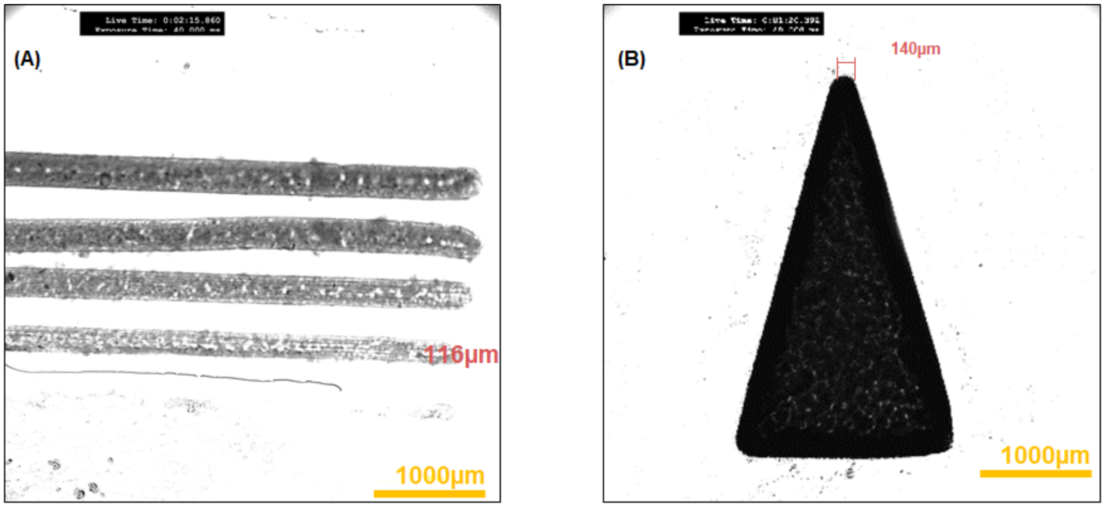

Supplementary Figure 1. A) Light-microscopy images of bar patterns for resolution analysis using 5% w/v PEDOT:PSS doped in 30% PEGDA for 0.6 UV exposure. B) triangle printed to demonstrate the *x–y* resolution. % v/v PEGDA**:**

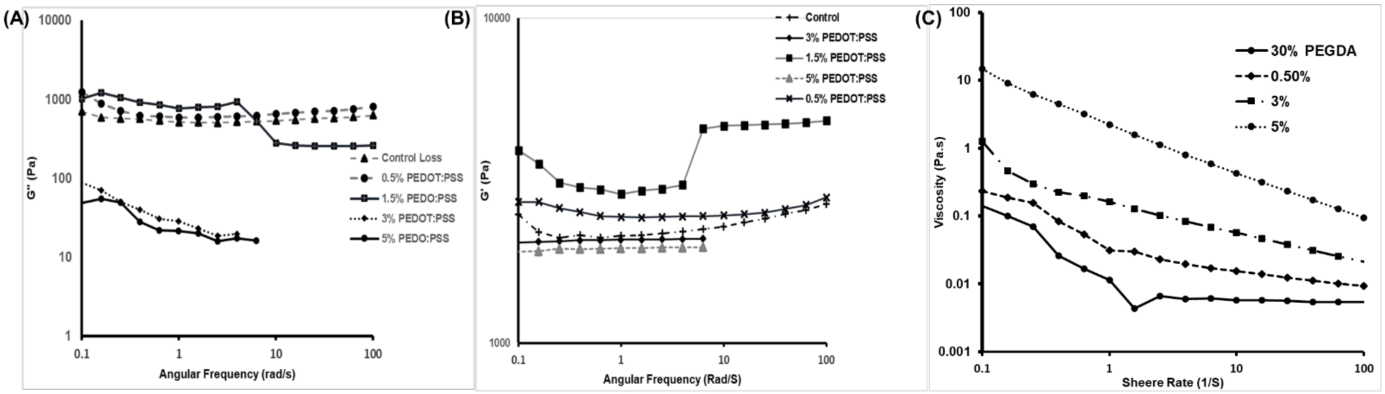

Supplementary Figure 2. Characterization of rheology performance of the PEDOT:PSS doped in 30% PEGDA. A, B) Shear storage moduli (G') and loss moduli (G'') of the DLP printed conducting polymer hydrogel inks (varying with PEDOT:PSS concentration) as a function of Frequency at 37 oC temperature. C) Viscosity versus shear stress for varying PEDOT:PSS concentration in 30% PEGDA at 25 oC

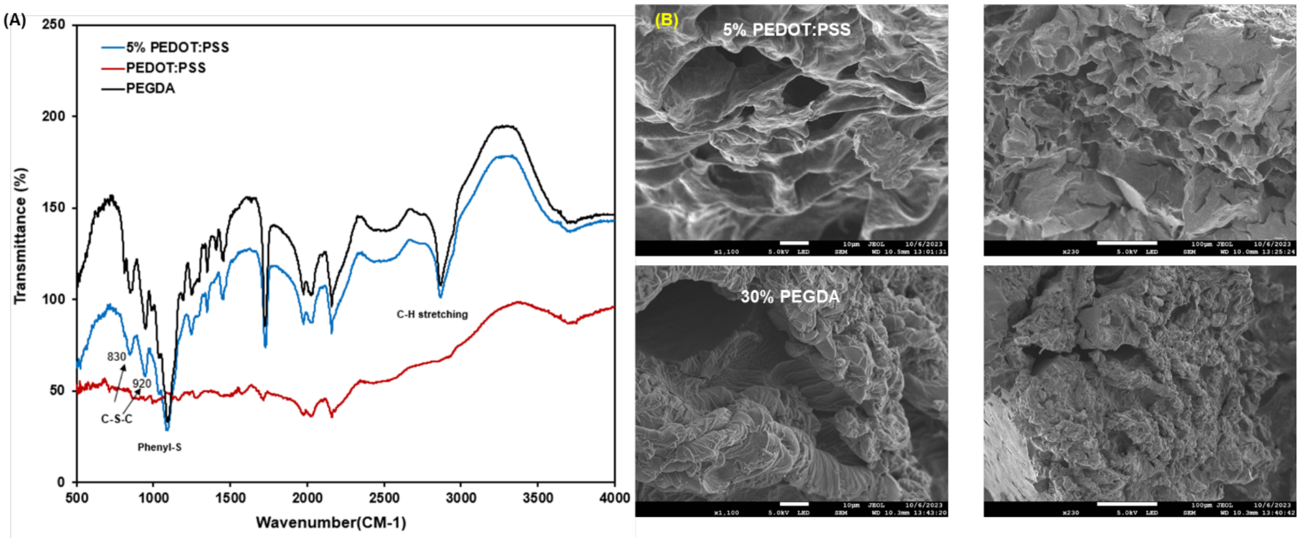

Supplementary Figure 3. A) FT-IR spectra of PEDOT:PSS and PEDOT:PSS doped in PEGDA hydrogel. B) Cross-sectional SEM images of lyophilized 5% PEDOT:PSS hydrogel and 30% PEGDA

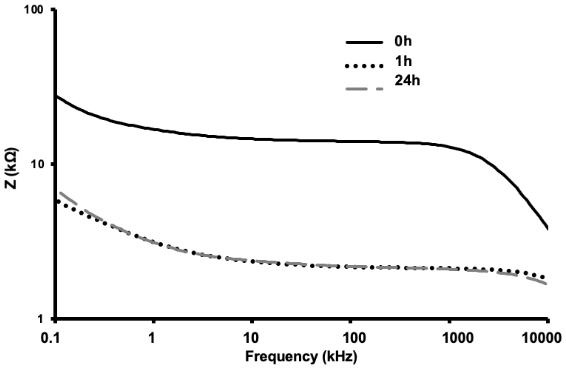

Supplementary Figure 4. Bode plot spectra of hydrogel-based electrodes to compare swelling of PEGDA between 5%-microparticle-doped-PEGDA**.**

(A)

(i)

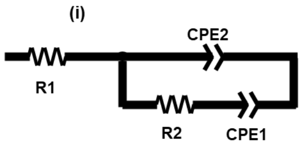

(ii)

Cell Density (Million cells/mL)	R_{ct}	CPE_{dl}	$n(CPE_{dl})$	CPE_{diff}	$n(CPE_{diff})$
0	23515.67	8.85E-12	0.95	1.8E-7	0.66
0.5	29445.67	7.09E-12	0.97	3.302E-6	0.57
1	37055.33	1.06E-11	0.93	1.469E-7	0.75
2	39749.67	4.32E-12	0.98	9.913E-8	0.645
3	47005.33	4.96E-12	0.99	1.195E-8	0.8

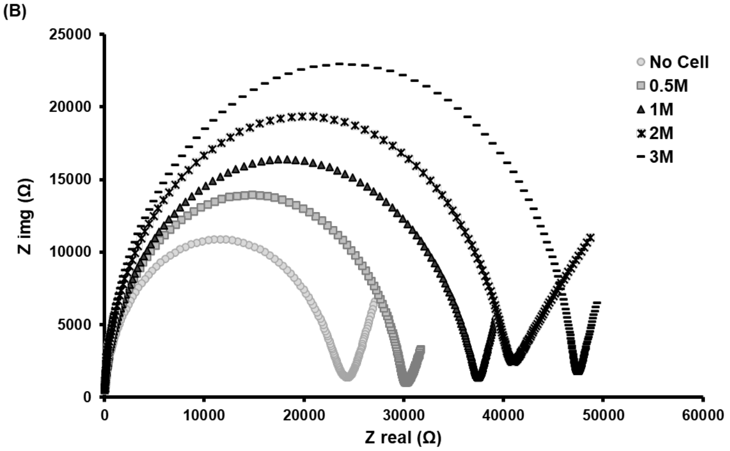

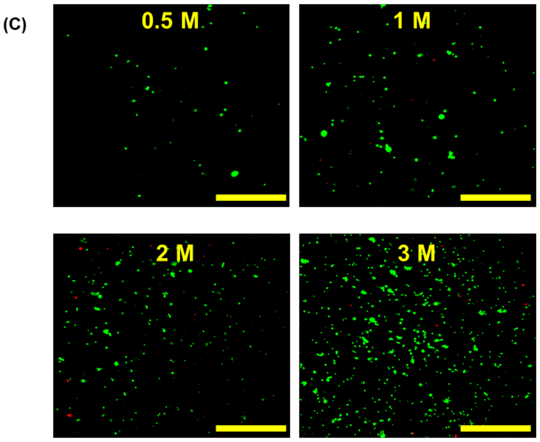

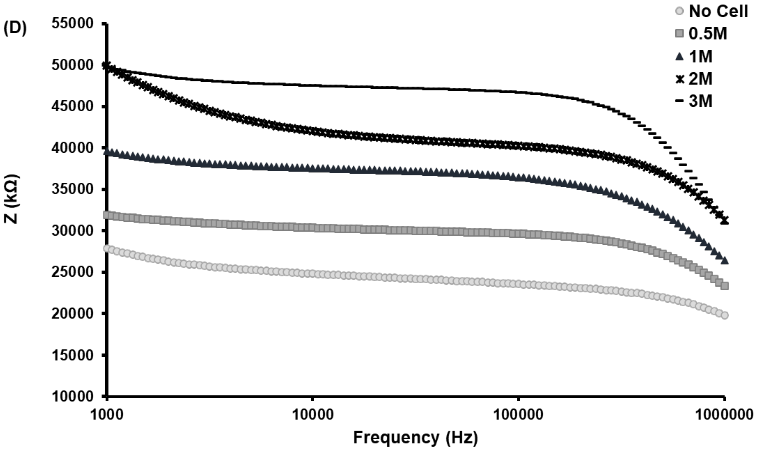

Supplementary Figure 5. A) EIS parameter analysis: i) Randles circuit is an equivalent electrical circuit, ii) Fitting parametric values of circuit elements in the equivalent circuit. B) Nyquist plot for cell-laden GelMA at different cell densities (0, 0.5, 1, 2, 3 million cells/mL), B) Live/dead imaging of cell-laden GelMA at different cell densities: 0.5, 1, 2, 3 million cells/mL. The scale bar is 200μm. D) Bode plot for cell-laden GelMA at different cell densities (0, 0.5, 1, 2, 3 million cells/mL).

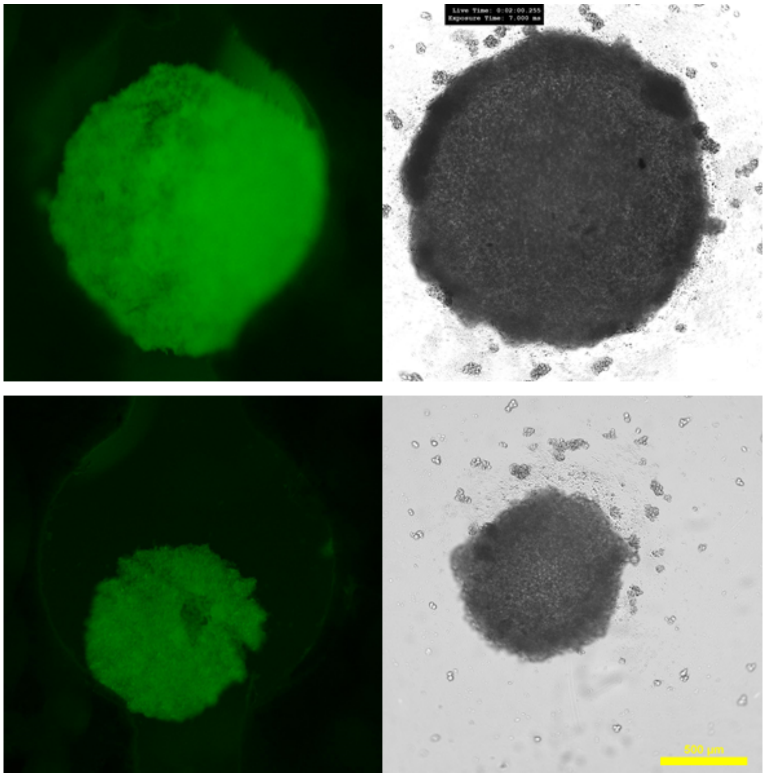

Supplementary Figure 6. A) Fluorescent image, B) Bright-field image: of 75k cells/ Spheroid of GFP-Tagged MDA-MB-231 (Spheroid Radius: 850μm). C)Fluorescent image, D) Bright-field image: of 75k cells/ Spheroid of GFP-Tagged MDA-MB-231 (Spheroid Radius: 425μm).

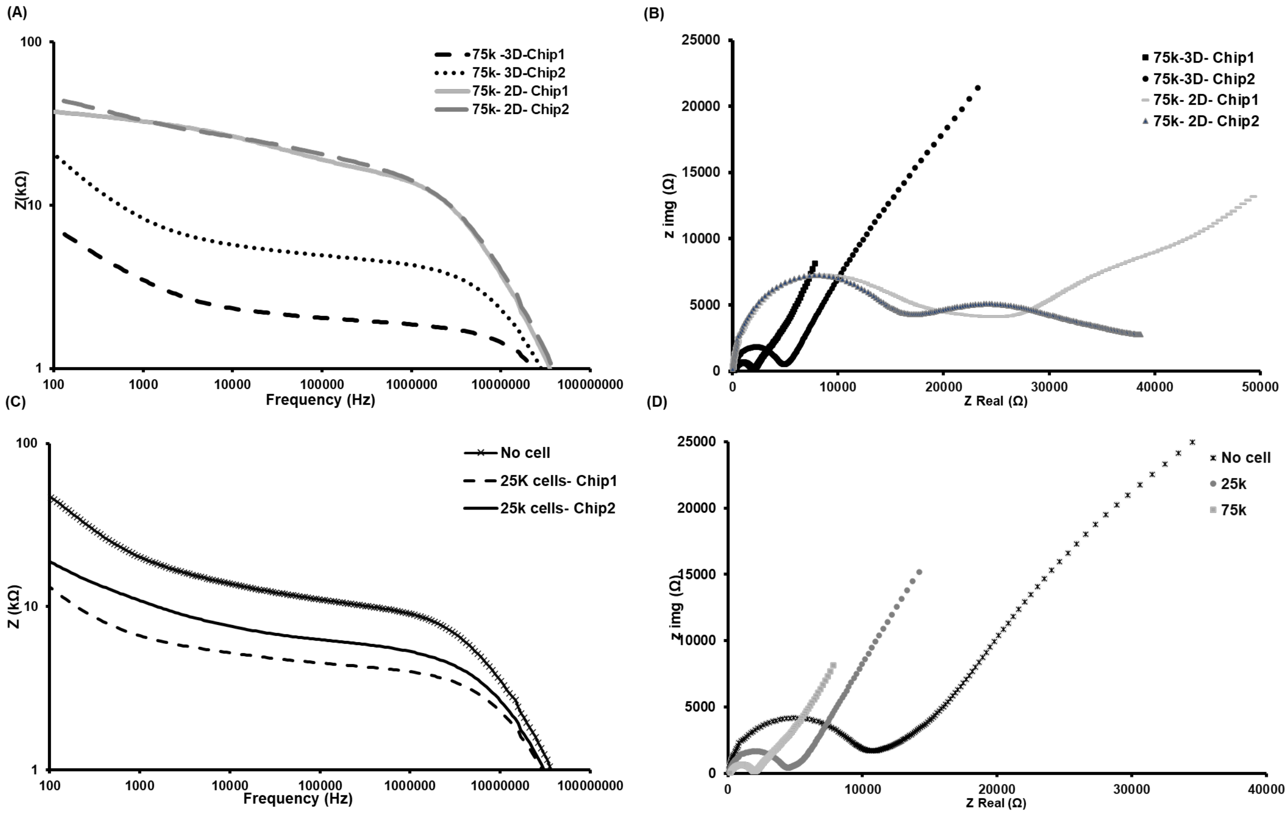

Supplementary Figure 7. A) Bode plot spectra of spheroid with and without GelMA matrix. B) Nyquist plot for spheroid with (3D) and without (2D) GelMA matrix. densities (75 cells/spheroid in 5% GelMA, C) Bode plot spectra of spheroid 25k cell encapsulated in GelMA matrix in comparison with GelMA with no spheroid. D) Nyquist plot for spheroid 25k cell encapsulated in GelMA matrix in comparison with GelMA with no spheroid. (25 cells/spheroid in 5% GelMA /mL).

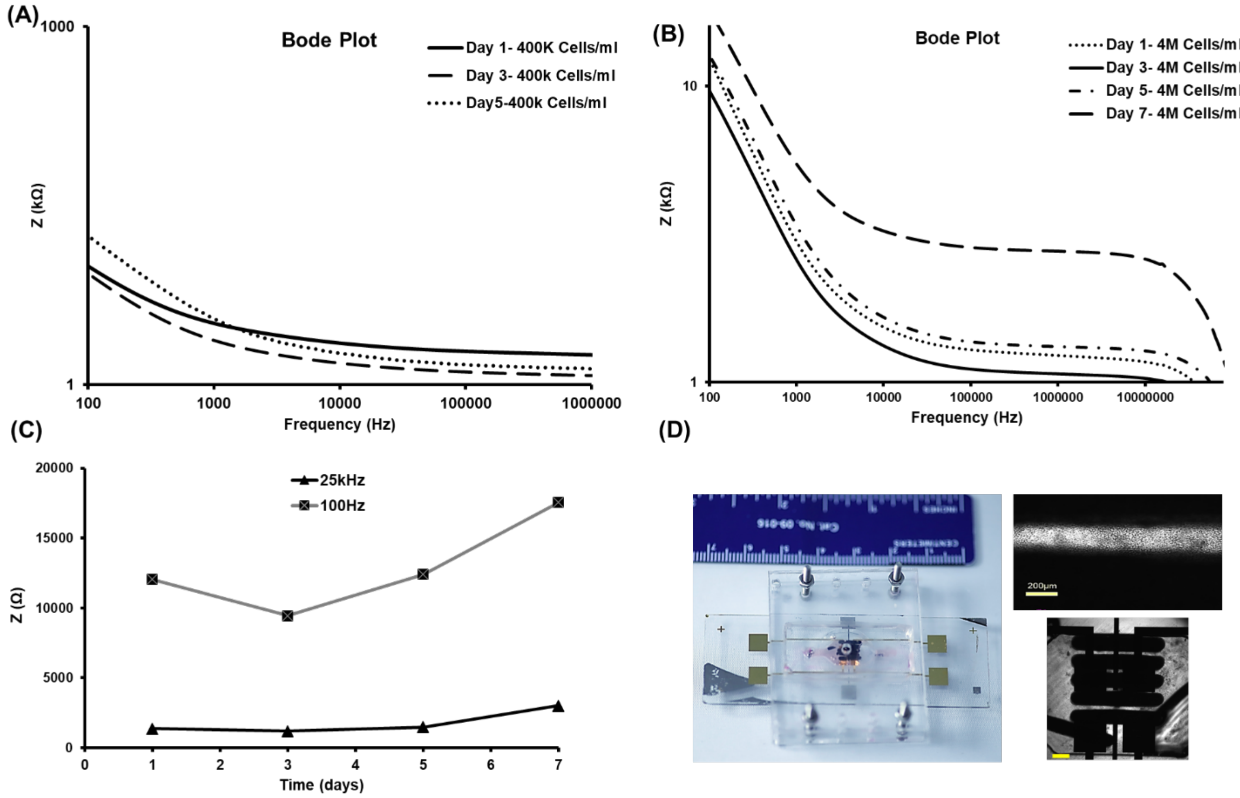

Supplementary Figure 8. A) Bode plot, time revolve EIS measurement during 5 days for 400k cell/mi in PEDOT:PSS microchannels B) Bode plot, time revolve EIS measurement during 5 days for 4M cell/mi in PEDOT:PSS microchannels, C) Time-revolved impedance magnitude in 100 and 25k Hz. D) Photographs showing electrical device design; hydrogel microchannels printed on Au electrodes(left); Brightfield microscopic images of the cells in printed channels (top right); printed PEDOT:PSS based microchannels on Au electrode glass slide (bottom right).
